# Temporal arteritis and vasculitic myopathy in polyarteritis nodosa

**DOI:** 10.1007/s00415-025-13199-6

**Published:** 2025-07-09

**Authors:** Nikolas Ruffer, Isabell Haase, Tingting Xiong, Florian Prinz, Daniel Koehler, Ina Kötter, Martin Krusche

**Affiliations:** 1 III. Department of Medicine, University Medical Center Hamburg-Eppendorf, Martinistraße 52, 20246 Hamburg, Germany; 2 Department of Rheumatology and Immunology, Klinikum Bad Bramstedt, Bad Bramstedt, Germany; 3 Department of Diagnostic and Interventional Radiology and Nuclear Medicine, University Medical Center Hamburg-Eppendorf, Hamburg, Germany

**Keywords:** Vasculitis, Myositis, Halo sign, PET/CT imaging, Tocilizumab

**Fig. 1:**
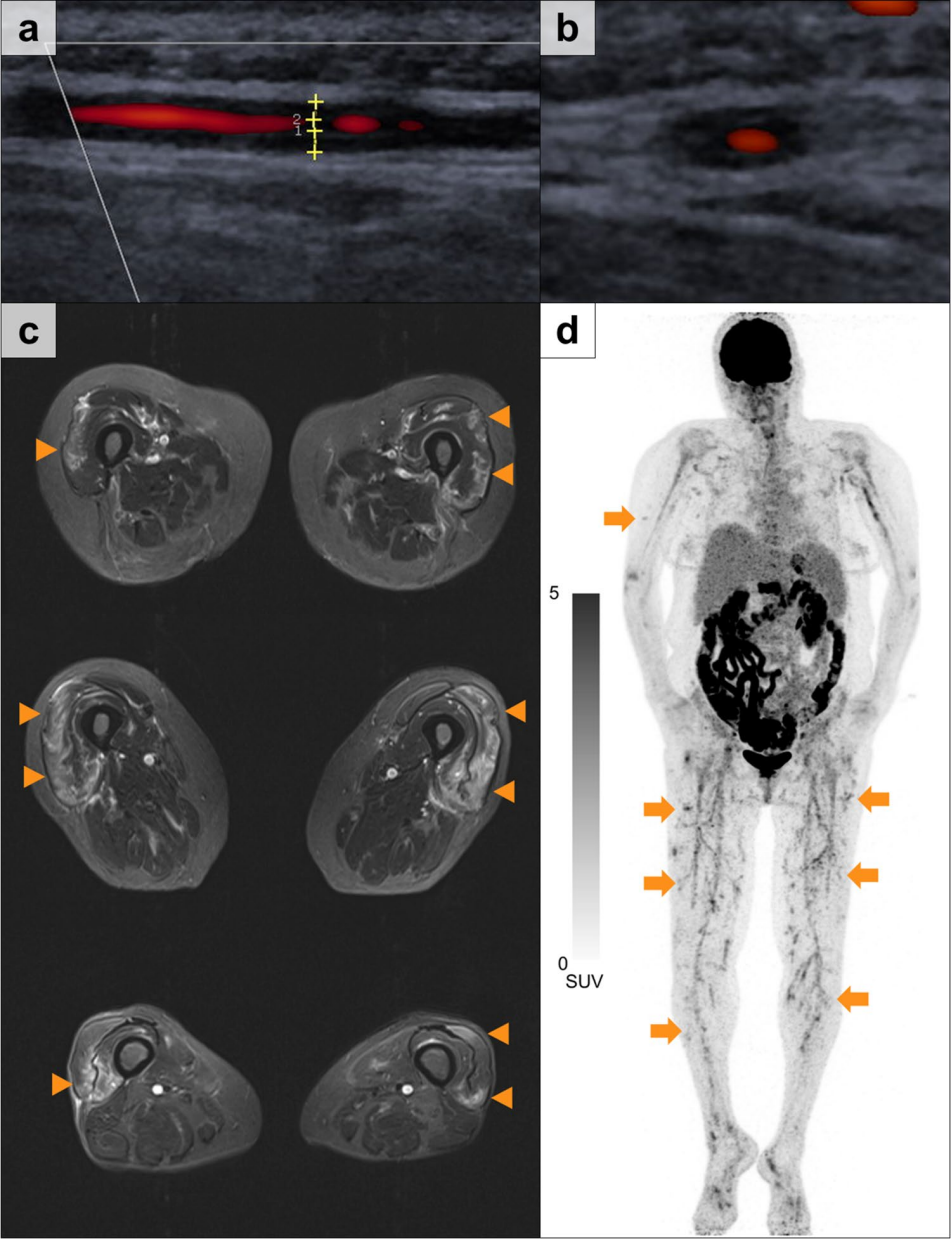
Imaging findings of temporal artery inflammation and vasculitic myopathy in systemic PAN. **a, b** Temporal artery ultrasound (longitudinal and axial plane) of the left frontal branch shows a non-compressible hypoechoic thickening of the arterial vessel wall (0.6 mm) consistent with a ‘halo sign’. **c** T2-weighted MRI (T2 TSE STIR, axial plane) findings of multifocal and patchy hyperintensities consistent with oedematous lesions secondary to vasculitis. **d** Corresponding whole body [^18^F]FDG PET/CT imaging demonstrates increased radiotracer uptake in peri- and intramuscular arteries (medium-sized vessels) pronounced in the lower extremities (arrows)

Dear Sirs,

A 61-year-old female presented with bilateral temporal headache, neck pain and myalgia of the thighs. On admission, physical examination was remarkable for a tender left temporal artery (TA) and symmetrical muscle weakness of the proximal lower extremities. However, vision was regular and skin changes or signs of arthritis were absent. Inflammatory markers (CRP 312.5 mg/L; normal < 5 mg/L) were highly elevated, while serum creatine kinase (CK) activity (17 U/L), creatinine and urine analysis were normal. ANCA testing was negative and neither myositis-specific antibodies nor anti-CCP antibodies were detected. Evaluation for viral infections (HBV, HCV, HIV) was negative. TA ultrasound demonstrated a bilateral non-compressible ‘halo sign’ consistent with TA inflammation (TAI) (Fig. 1a). In addition, T2-weighted MRI of both thighs revealed signs of fasciitis and patchy areas of increased signal intensity within the musculature (Fig. 1c). Whole body [^18^F]FDG PET/CT imaging was carried out to evaluate a systemic vasculitic disorder. Corresponding to the MRI signal alterations, an increased uptake of FDG in medium-sized vessels that was pronounced in the musculature of thighs, was noted (Fig. 1b). Signs of large vessel vasculitis such as aortitis were absent. Based on these findings, a diagnosis of systemic polyarteritis nodosa (PAN) was made. Subsequent introduction of glucocorticoid therapy and tocilizumab induced clinical remission in this patient [8, 9].

PAN is a systemic necrotizing vasculitis that frequently presents with vasculitic myopathy (VM) and concomitant fasciitis. ‘MRI myositis sine myositis’ [1] represents the typical finding of VM and refers to normal serum CK activity in the presence of diffuse or patchy hyperintensities on T2-weighted MRI that resemble myositis. However, the distinction of VM and myositis is of great interest to the clinician because these entities differ in pathophysiology, organ involvement and, therefore, management. For example, tocilizumab is not suited for the treatment of myositis in most clinical situations but achieves high rates of remission in PAN [6].

In general, VM of the lower extremity is virtually non-existent in giant cell arteritis (GCA) and most likely indicates PAN or ANCA-associated vasculitis [2, 11].

The diagnostic utility of [^18^F]FDG PET/CT imaging in PAN recently gained attention since a pattern of increased radiotracer uptake in skeletal muscle tissues and intramuscular arteries (so-called ‘dirty muscle sign’) has been described as a specific finding of VM in PAN [5, 10].

Also, PAN can rarely present with signs of TAI and these cases may mimic giant cell arteritis [4]. Notably, an association of VM (including fasciitis) and TAI has been reported in a TA biopsy study of PAN patients [7] and one prospective TA ultrasound study [3]. However, systematic studies investigating TAI in PAN have not been conducted to the best of our knowledge.

In conclusion, our case highlights the classic presentation of VM that may be misdiagnosed as ‘myositis’. VM is prototypically characterized by myalgia, normal serum CK activity and diffuse or patchy hyperintensities on T2-weighted MRI [2, 11]. Finally, TAI is not pathognomonic for GCA and careful interpretation of additional clinical findings guides the diagnosis of GCA mimickers such as PAN.
